# Combined immunohistochemistry and FISH testing for diagnosing metaplastic thymoma: a case report of misdiagnosis as pulmonary mucinous epidermoid carcinoma

**DOI:** 10.3389/fsurg.2026.1837583

**Published:** 2026-06-24

**Authors:** Jiaxin Liu, Xiang Yu, Wenquan Li, Chunhui Shen, Zhenhui Wang, Jiyong Wang

**Affiliations:** 1 The First Clinical Medical College of Guangzhou University of Chinese Medicine, Guangzhou, China; 2 Department of Thoracic Surgery, First Affiliated Hospital of Guangzhou University of Chinese Medicine, Guangzhou, China

**Keywords:** differential diagnosis, FISH testing, immunohistochemis try, pulmonary mucinous epidermoid carcinoma, thymoma of metaplastic origin

## Abstract

**Simple summary:**

Differentiating metaplastic thymoma from pulmonary mucoepidermoid carcinoma through immunohistochemistry and FISH testing provides diagnostic evidence for clinical practice. This study investigates the clinical-pathological features, immunophenotype, and di agnostic criteria of metaplastic thymoma (MT), distinguishing it from pulmonary mucinous epidermoid carcinoma (PEMC).

**Methods:**

Clinical data from a single patient with surgically confirmed metaplastic thymoma were collected. Histological examination, immunohistochemical staining, and molecular testing were performed.

**Results:**

The patient was a middle-aged woman. Postoperative pathological immunohistochemistry revealed a tumor composed of relatively benign epithelioid and spindle cells. Epithelioid cells exhibited nests with mildly enlarged nuclei and rare mitotic figures, accompanied by focal degenerative necrosis. Immunohistochemistry results: CD117 (–), CD20 (–), CD3 (speckled +), CD34 (–), CD5 (speckled +), CD99 (–), CK20 (–), CK7 (partial +), EMA (spindle cells +; epithelioid cells speckled perinuclearly +), Ki67 (5%+), P53 (80%+), P63 (+), Syn (-), TDT (-). Molecular testing: YAP1-MAML2 gene break-up rearrangement detected.

**Conclusion:**

Metaplastic thymoma represents a rare subtype of thymoma. Diagnosis requires integration of clinical presentation, microscopic morphology, immunohistochemical features, and molecular biology data to enhance diagnostic accuracy and facilitate effective differentiation from pulmonary mucinous epidermoid carcinoma.

## 1 Introduction

Metaplastic thymoma (MT) is a rare thymoma accounting for less than 1% of all thymoma types. Histologically, it consists of nests of epithelial cells distributed among fragile spindle cells (1), exhibiting biphasic differentiation. Clinically, MT typically does not present with signs of myasthenia gravis, unlike other thymoma types that may cause vascular injury, postoperative myasthenic crisis, pain, and increased infection risk (2). Pulmonary mucoepidermoid carcinoma (PMEC) is a rare primary salivary gland-type malignancy of the lung, accounting for less than 1% of all thoracic tumors. It originates from Kulchitsky's cells within the submucosal glands of the trachea and bronchi. According to the WHO definition, PMEC is a malignant epithelial tumor composed of mucin-producing cells, squamous cells, and intermediate cells, exhibiting solid, glandular, or cystic patterns (3).

This report describes a case of metaplastic thymoma misdiagnosed as pulmonary mucoepidermoid carcinoma. Through systematic analysis of the lesion's clinical-pathological features, immunohistochemical phenotype, and molecular testing results, key differential diagnostic points from PMEC were clarified, thereby providing reference guidelines and practical insights for the clinical management of MT.

## 2 Materials and methods

### 2.1 Case records

The patient, a female, is from the First Affiliated Hospital of Guangzhou University of Chinese Medicine and was admitted due to recurrent chest tightness lasting more than two months. Initially, the symptoms manifested as occasional chest tightness following physical activity, which resolved with rest. Over the past month, the symptoms have gradually worsened; they now occur even at rest, with increased severity and a duration of approximately 10–30 min per episode. The chest tightness was described as a sensation of pressure, localized to the lower sternum and the left anterior chest, with no radiating pain. There were no accompanying symptoms of dyspnea, palpitations, fever, cough, sputum production, hemoptysis, weight loss, or night sweats. The patient explicitly denied a history of recent trauma, radiation therapy, or travel.

**Figure 1:**
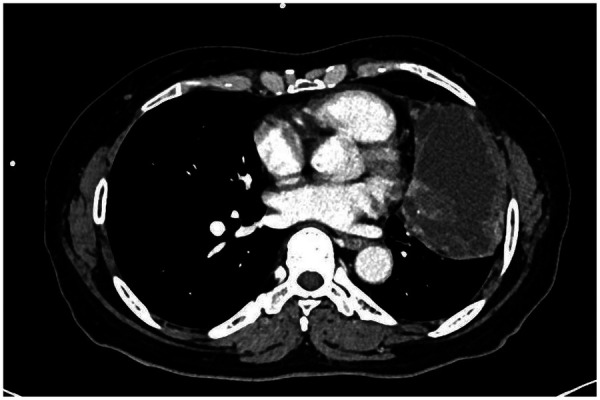
Contrast-enhanced chest CT (mediastinal window, 1.25 mm slice thickness) showing a heterogeneously enhancing mass in the left lung (arrow).

A chest contrast-enhanced CT scan (as shown in Figure 1) performed at another hospital revealed a cystic-solid mass in the left lower lobe, initially suspected to be a pulmonary blastoma. PET-CT imaging demonstrated a large soft tissue mass (with localized necrosis) in the left anterior-inferior mediastinum, exhibiting significantly elevated metabolic activity, consistent with a mesenchymal malignancy. A percutaneous transpulmonary biopsy was performed. Pathology confirmed non-small cell lung cancer (NSCLC) with clinical TNM staging of cT4N0M0, stage IIIA. Further histological examination of the left lung biopsy specimen revealed dense, solid, and patchy distributions of spindle-shaped and oval cells within the pulmonary parenchyma. The cells exhibited a mild cytomorphology with few mitotic figures. Based on morphology and immunohistochemical staining, mucinous epidermoid carcinoma was considered. Immunohistochemistry results: TIF1 (–), CK (+), P40 (+), VIN (–), CD34 (–), STAT6 (–), Bc12 (+), CD99 (–), Des (–), SMA (+), S-100 (–), ALK (+), CR (–), Ki67 index approximately 5%. The patient initially received a combination chemotherapy and immunotherapy regimen: Toripalimab 240 mg + Carboplatin 500 mg + Albumin-bound Paclitaxel 400 mg. This synergistic approach aimed to control tumor progression. Following initial treatment, the patient transitioned to oral targeted therapy with Anlotinib 12 mg (Days 1–12). Assessment indicated suboptimal response to this regimen, with tumor control falling short of the anticipated goals.

### 2.2 Treatment approach and outcomes

Due to poor response to prior therapies, our thoracic surgery team performed a thoracoscopic mediastinal mass resection, which was converted to open exploration during the procedure. Postoperative pathological confirmation was achieved through immunohistochemistry and fluorescence *in situ* hybridization techniques. Possible reasons for the misdiagnosis at the previous hospital included the tumor's large size, absence of visible metastases, and poor treatment response. These factors may stem from initial diagnostic bias or inaccurate biopsy sampling, necessitating consideration of potential lesions such as giant thymoma.

#### 2.2.1 Intraoperative findings and microscopic examination

**Figure 2:**
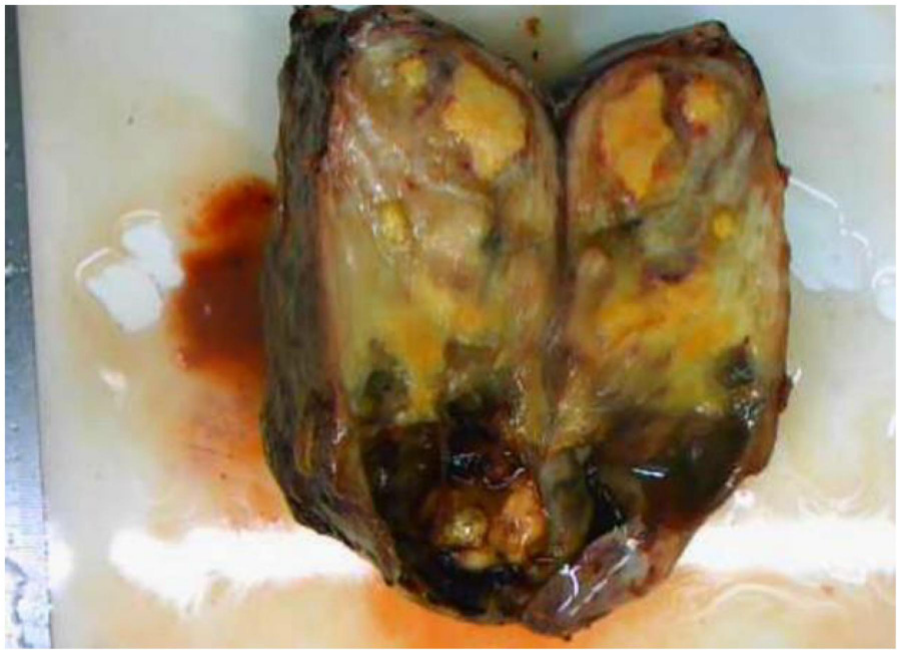
Gross appearance of the resected tumor, demonstrating a solid mass with central necrosis (scale bar: 1 cm).

**Figure 3:**
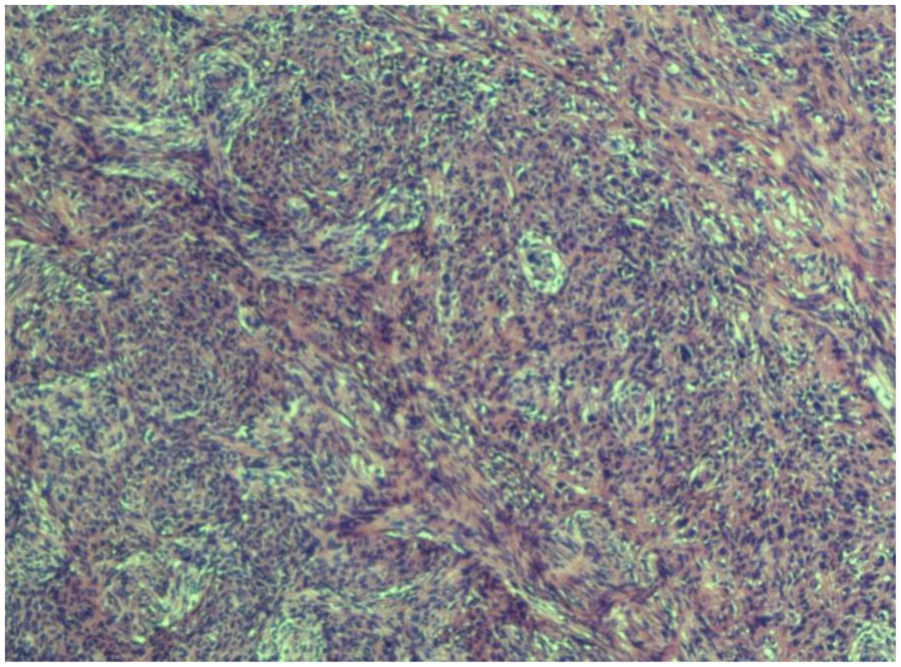
Histopathology of the tumor (HE stain, ×40 magnification) showing atypical spindle cells consistent with a high-grade malignant neoplasm.

Macroscopic Findings: Mediastinal lymph nodes and mediastinal mass measuring 14 cm × 11 cm × 4 cm (Figure 2). Microscopic Findings: Mildly differentiated epithelioid and spindle cells were observed. Epithelioid cells exhibited nest-like arrangements with mildly enlarged nuclei and rare mitotic figures, accompanied by focal degeneration and necrosis (Figure 3).

#### 2.2.2 Immunohistochemistry

Immunohistochemistry results: CD117 (–), CD20 (–), CD3 (speckled +), CD34 (–), CD5 (speckled +), CD99 (–), CK20 (–), CK7 (partial +), EMA (spindle cells +; epithelioid cells speckled+perinuclearly), Ki67 (5%+), P53 (80%+), P63 (+), Syn (-), TDT (-).

#### 2.2.3 FISH analysis

**Figure 4:**
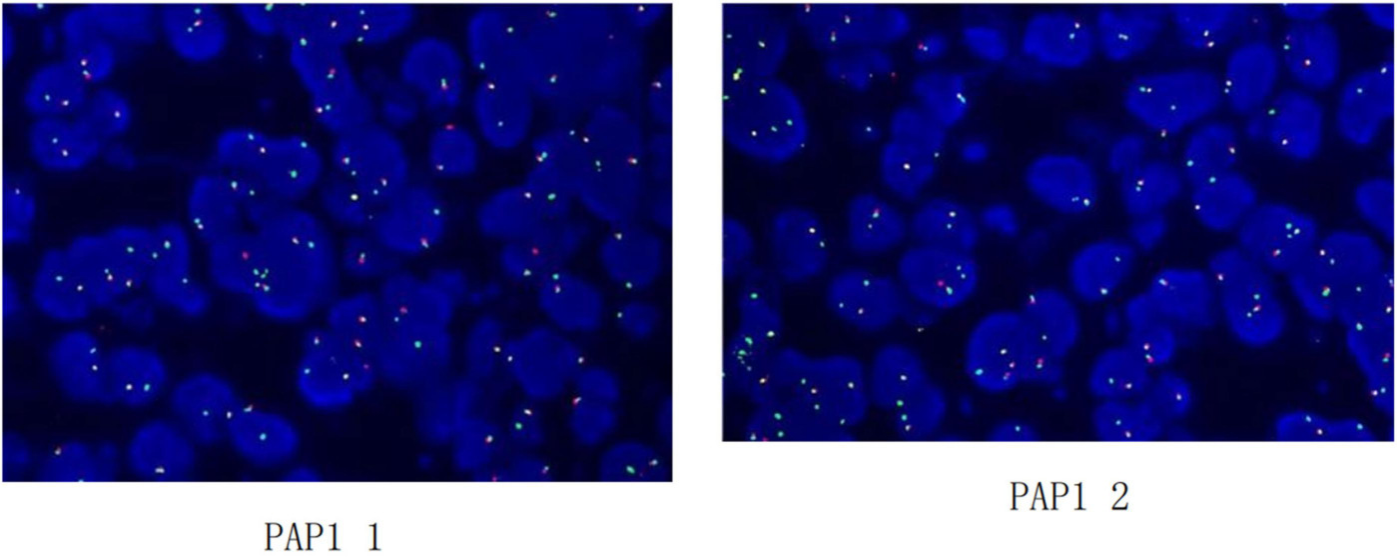
Indicates a deletion of the YAP1 gene (red signal). The number of tumor cells evaluated was 200. Normal fluorescence signals consist of two adjacent red and two adjacent green signals, or two yellow signals. Abnormal signals include one red, one green, and one yellow signal; two red and two green signals separated; and other types of separated red-green signals. The breakage ratio was 2%.

**Figure 5:**
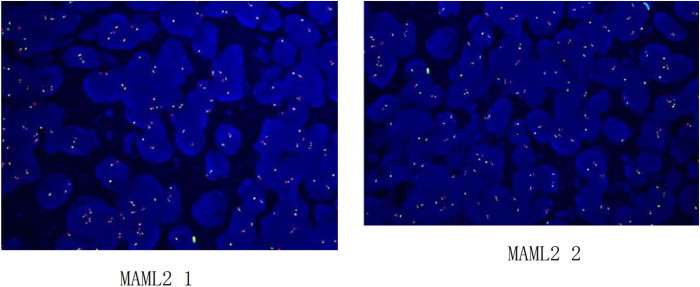
Conclusion indicates MAML2 gene breakage (present). Number of tumor cells used for estimation: 200. MAML2 gene breakage: Normal fluorescence signal appears as two adjacent red and two adjacent green signals, or two yellow signals. Abnormal signal appears as one red, one green, one yellow, or two red signals. Criteria: MAML2 gene breakage ratio >10% is positive; ≤10% is negative. Separation of two green signals or other types of red-green signal separation. Breakage ratio: 28%.

φ YAP1 FISH Result: Breakage ratio 2%, detecting YAP1 gene (red signal) deletion (as shown in Figure 4). Note: This specimen exhibits YAP1 gene abnormality in FISH results. *κ* MAML2 FISH Result: FISH result (+), MAML2 gene (present) breakage (as shown in Figure 5).

#### 2.2.4 Clinical reasoning and diagnostic tests

Preoperative laboratory tests revealed mild anemia (hemoglobin 92 g/L, reference range 115–150; red blood cell count 2.95 × 10^12/L, reference range 3.8–5.1). Liver and kidney function were essentially normal (ALT 8 U/L, AST 20 U/L, creatinine 75 μmol/L), significantly elevated CA19-9 (120.3 U/mL, reference range 0–27), and elevated D-dimer (2.72 mg/L, reference range 0–0.55). and elevated fibrinogen (4.61 g/L, reference range 2.0–4.0), suggesting a hypercoagulable state or tumor-associated inflammatory response. High-sensitivity thyroid-stimulating hormone (TSH) was 5.437 mIU/L (reference range 0.560–5.910, at the upper limit of normal); free T3/T4 were normal, with no clinical manifestations of hypothyroidism, and no intervention was performed. CRP, ESR, and white blood cell count were normal, not consistent with active infection. Symptoms suggestive of myasthenia gravis had been ruled out preoperatively, and acetylcholine receptor antibody testing was negative. Intraoperatively, the tumor was found to be tightly adherent to the left lower lobe of the lung and the chest wall, making dissection difficult; therefore, the procedure was converted to open thoracotomy for exploration, and the mass was completely resected. Given the ineffectiveness of systemic treatment at the referring hospital, the tumor's confinement to the thoracic cavity, and the absence of distant metastasis, the multidisciplinary team (MDT) unanimously agreed that complete surgical resection was the optimal curative approach. The timely conversion to open thoracotomy during surgery, based on the extent of adhesions, prevented bleeding or tumor rupture that might have resulted from endoscopic dissection. Postoperative pathology confirmed a metaplastic thymoma with benign biological behavior; therefore, adjuvant radiotherapy or chemotherapy was not required.

Our hospital's thoracic surgery team performed the radical resection, and the postoperative specimen was sent for histopathological and FISH analysis. The immunohistochemistry and FISH results corrected the previous hospital's diagnosis of “mucinous epidermoid carcinoma” or “non-small cell lung cancer.” Based on the morphological and molecular findings, the final histopathological diagnosis was “metaplastic thymoma.” Postoperative follow-up chest x-rays showed no signs of recurrence. The patient was treated with cefuroxime for infection, flurbiprofen for pain, and nebulization for symptomatic support. The chest drainage tube was removed on the fifth postoperative day. The percutaneous lung biopsy performed at the previous hospital may have been limited to the solid components of the tumor, failing to capture the complete histological features of the cystic regions; Furthermore, metaplastic thymoma is rare and may present as a large soft tissue mass with increased metabolism. Combined with the lack of MAML2 gene rearrangement testing, this led to an initial misdiagnosis. Clinically, it must be differentiated from pulmonary mucoepidermoid carcinoma (which is typically characterized by MAML2 rearrangement negativity, although MAML2 positivity in this case supports the diagnosis of thymoma). In this case, the aforementioned diagnosis was ultimately ruled out, and the final diagnosis was metaplastic thymoma. A bronchoscopy was performed on the third postoperative day. Endoscopic examination revealed smooth mucosa in the basal segment of the left lower lobe, with patent airways and no obvious neoplasms. Cytological examination of the bronchoalveolar lavage fluid showed no malignant cells, and bacterial/fungal cultures were negative, ruling out anastomotic infection or empyema. The patient is currently recovering well, and a follow-up enhanced chest CT scan and CA19-9 monitoring are scheduled one month postoperatively.

## 3 Differential diagnosis

Both MT and PMEC are clinically rare diseases, with nonspecific clinical manifestations and imaging features. Diagnosis currently relies primarily on postoperative histopathological evaluation, though the complexity of histological components poses significant diagnostic challenges. Accurate diagnosis hinges on integrating immunohistochemistry with FISH testing, focusing on specific markers such as CD series molecules and YAP1-MAML2 gene fusions. This approach, combined with systematic analysis of histological features, enables more reliable differentiation between MT and PEMC. In clinical practice, multidisciplinary collaboration integrating comprehensive assessment of imaging, pathology, and clinical presentation can effectively reduce the risk of misdiagnosis (See Table 1 for details).

**Table 1:** Differential diagnosis of MT and PMEC.

Characteristics	MT	PMEC
Clinical Features	It commonly occurs in the anterior mediastinum. Clinically, most patients are asymptomatic, with the condition often discovered incidentally during physical examinations and without noticeable discomfort. Occasionally, patients may experience symptoms such as chest pain, cough, or shortness of breath.	Smaller tumors may present with no obvious symptoms. Tumors located in the segmental bronchi or higher may manifest as bronchial irritation and obstruction symptoms (19).
Key pathological indicators	YAP1-MAML2 or YAP1-NUTM1 fusion.MAML2 gene status: no break (or fused with YAP1, but detectable as a break by FISH).	CRTC1-MAML2 fusion The MAML2 gene exhibits a breakpoint (resulting in CRTC1-MAML2)
Organizational Structure	Encapsulated, cut surface grayish-white or grayish-yellow, solid, moderately firm. Microscopically composed of epithelioid and spindle cells. Epithelioid cells arranged in island-like or trabecular patterns. Cells polygonal or oval, nuclei oval. Mild to moderate cellular atypia may be present.	Low-grade cancers predominantly exhibit cystic components with no significant cellular abnormalities, and most lack areas of necrosis, though calcifications may be observed. High-grade cancers reveal atypical cells, mitotic figures, necrosis, and local lymph node metastasis.
Immunophenotype and Molecular Genetics	Epithelial cells show diffuse positivity for CK, CK5/6, and E-cadherin; CK19 and EMA expression is variable; vimentin is negative. Spindle cells demonstrate diffuse vimentin positivity, focal EMA positivity, and E-cadherin negativity. Ki-67 proliferation index <5%. Background lymphocytes were positive for CD3, CD5, and CD20, and negative for TDT (20).	CK7, Muc5AC, p63, and p40 were positive; AB/PAS staining was positive (a specific marker for mucus); TTF-1 and Napsin A were negative. The Ki-67 index serves as a pathological auxiliary diagnostic indicator for PMEC, with P40 exhibiting lower sensitivity than p63. The Ki-67 index ranged from 2% to 80%.
Imaging Findings	A chest CT scan revealed a well-defined, round or oval-shaped solid mass located in the anterior mediastinum.	When a chest CT reveals lesions located within the proximal bronchi and growing along the bronchial lumen, or when accompanied by obstructive pneumonia.

### 3.1 Pulmonary mucinous epidermoid carcinoma

PMEC is a tumor composed of mucinous cells, epidermoid cells, and intermediate cells (4). This disease has a low incidence and lacks specific clinical signs or imaging features. When CT scans reveal pulmonary masses—particularly lesions located within proximal bronchi, growing along the airway lumen, or associated with obstructive pneumonia—this diagnosis should be considered (3). Symptoms primarily depend on the tumor's anatomical location and size. Smaller or peripherally located tumors often cause no significant clinical symptoms. Tumors in segmental bronchi or proximal airways may induce bronchial irritation or obstruction-related symptoms, including cough, sputum production, hemoptysis, chest pain, chest tightness, and fever (5).

PMEC can be classified as high-grade or low-grade based on pathological findings. Low-grade carcinoma is more common, characterized by predominant cystic components, typically lacking areas of necrosis, and often exhibiting calcifications. High-grade cases may reveal atypical cells, mitotic figures, necrosis, and local lymph node metastasis (6). Common immunohistochemical markers in PMEC pathology include CK7, Muc5AC, p63, and p40 positivity. AB/PAS staining demonstrates mucin-specific positivity, while TTF-1 and Napsin A are negative. The Ki-67 proliferation index serves as an auxiliary indicator, aiding in distinguishing squamous cell carcinoma, adenocarcinoma, and adenoid cystic carcinoma. p63 is frequently used to differentiate mucinous epidermoid carcinoma from other salivary gland tumors. p40 exhibits a similar expression pattern to p63 but with slightly lower sensitivity. Ki-67 detection ranges from approximately 2% to 80%. The average Ki-67 index for high-grade PMEC is about 22.4%, while low-grade PMEC averages around 4.1%. This difference holds some reference value for the grading diagnosis of PMEC (7). Diagnosing PMEC requires the presence of three cell types: mucinous epidermoid cells, epidermoid cells, and intermediate cells. Additional supportive immunohistochemical and cytochemical staining techniques, along with MAML2 translocation analysis, can aid diagnosis (4). Currently, the CSCO Head and Neck Cancer Diagnosis and Treatment Guidelines recommend FISH testing for MAML2 gene rearrangement as a key molecular marker for PMEC (8). PMEC shares significant histological similarities with mucinous epidermoid carcinoma (MEC) arising in other sites. While anatomical location differences may influence tumor biology, both share common pathological features in terms of genetic abnormalities. Literature reports (9) indicate that MAML2 gene rearrangement correlates with prognosis in PEMC patients, with CRTC1-MAML2 fusion gene-positive patients exhibiting longer survival and lower recurrence/metastasis rates. Current PMEC treatment protocols may reference non-small cell lung cancer approaches, with common clinical interventions including chemotherapy, radiotherapy, and immunotherapy or targeted therapy.

### 3.2 Metaplastic thymoma

Metaplastic thymoma is a rare thymic tumor predominantly occurring in the anterior mediastinum, with extramedullary presentation being uncommon. It exhibits a higher incidence in females. Hou Weidong et al. (10) reported a case of metaplastic thymoma originating within the thyroid gland. CT imaging of metaplastic thymoma (MT) typically reveals a round, oval, or irregularly shaped solid mass in the anterior mediastinum with well-defined borders. The lesion exhibits uniform or slightly hypointense density on non-contrast imaging and shows mild to moderate enhancement after contrast administration. PET-CT demonstrates increased FDG metabolism with elevated SUV values, resembling thymic carcinoma. MT is a rare thymic tumor subtype originating from thymic epithelial cells. Other literature terms it as sarcomatoid interstitial thymoma, biphasic thymoma, low-grade metaplastic carcinoma, or mixed pleomorphic and spindle cell thymoma (11). Macroscopically, the tumor typically exhibits an intact capsule with a grayish-white or grayish-yellow cut surface and moderate consistency as a solid mass. Microscopically, it consists of epithelioid and spindle cells arranged in island-like or trabecular patterns. Cells are predominantly polygonal or oval with oval nuclei, exhibiting mild to moderate atypia (12). When mucoepidermoid carcinoma predominantly consists of spindle cells and squamous cells with inconspicuous mucin-producing cells, its morphology becomes difficult to distinguish from MT.

Immunohistochemical features: Epithelial cells show diffuse positivity for CK, CK5/6, and E-cadherin; CK19 and EMA expression is variable; vimentin is negative. Spindle cells demonstrate diffuse vimentin positivity, focal EMA positivity, and E-cadherin negativity. The Ki-67 proliferation index is low (<5%). Background lymphocytes show positivity for CD3, CD5, and CD20, while TDT is negative. Occasionally, a small number of TDT-positive lymphocytes are observed. Only in the case reported by Tajima were numerous TDT-positive lymphocytes seen surrounding epithelial cell nests (13). Clinically, MT cases are relatively rare, and literature evidence is insufficient. Its pathogenesis has undergone a lengthy research process. Zhao et al. (14) discovered YAP1-MAML2 gene fusions in some MT patients during their research. Marina et al. (15) first reported recurrent genetic features of YAP1-MAML2 fusion in MT. This fusion generates a protein that promotes tumor growth by inducing YAP1-related transcriptional signaling through a TEAD1-dependent mechanism (16). Although YAP1-MAML2 fusion is considered a potential common mechanism in MT, its limited case numbers warrant further investigation (17). Both epithelial markers (EMA and CKpan) show varying degrees of positivity in the two components of MT, suggesting both epithelial and spindle cells constitute tumor components, with spindle cells potentially arising from epithelial-mesenchymal transition (12).

Surgical resection remains the primary treatment for MT (18). Complete surgical removal of MT yields favorable prognosis with rare recurrence.

## 4 Conclusion

The key to differentiating MT from PMEC lies in the presence of the MAML2 fusion. However, due to varying associated genes, definitive diagnosis requires FISH testing and NGS. MT exhibits clinical insidiousness and represents a tumor with low malignant potential; early diagnosis and treatment are crucial (21). Clinically, imaging localization, histopathological features, and immunological/molecular testing should be integrated to prevent misdiagnosis. A comprehensive diagnosis should guide the development of appropriate surgical and follow-up treatment plans. Through multidimensional evaluation encompassing morphology, immunology, molecular biology, and imaging, accurate differentiation between MT and PMEC can be achieved, thereby enhancing diagnostic and therapeutic quality.

## Data Availability

The raw data supporting the conclusions of this article will be made available by the authors, without undue reservation.
